# Metabolome integrated with transcriptome reveals the mechanism of three different color formations in *Taxus mairei* arils

**DOI:** 10.3389/fpls.2024.1330075

**Published:** 2024-01-23

**Authors:** Yadan Yan, Yafeng Wen, Ye Wang, Xingtong Wu, Xinyu Li, Chuncheng Wang, Yanghui Zhao

**Affiliations:** ^1^ Central South University of Forestry and Technology, Changsha, Hunan, China; ^2^ Hunan Big Data Engineering Technology Research Center of Natural Protected Areas Landscape Resources, Changsha, China; ^3^ Yuelushan Laboratory Carbon Sinks Forests Variety Innovation Center, Changsha, China

**Keywords:** Maire yew, aril color, flavonoid, carotenoid, differentially expressed genes, transcription factors

## Abstract

Maire yew (*Taxus mairei*), an evergreen conifer, has high ornamental and medicinal value. The arils of this species has three different colors. However, the variation mechanisms of arils color formation remains unclear. Here, the gene expression and metabolite concentration were profiled for red (RTM), yellow (YTM), and purple (PTM) arils in different developmental stages. A total of 266 flavonoids and 35 carotenoids were identified. The predominant pigments identified in YTM were epiafzelechin, lutein, and β-Cryptoxanthin, while malvidin-3,5-di-O-glucoside and apigenin played crucial roles in PTM. And significant differential expression was observed among the *HCT*, *DFR*, *LAR*, *ANS*, *crtB*, *NCED*, and *CCoAOMT* genes across different color arils. During the maturation of yellow arils, the upregulation of *HCT* was strongly correlated with the accumulation of epiafzelechin. The diminished expression of *DFR*, *LAR*, and *ANS* seemed to inhibit the production of delphinidin-3-O-rutinoside. The decrease in *crtB* expression and concurrent increase in *NCED* expression potentially regulate the heightened accumulation of lutein. Meanwhile, the accumulation of β-cryptoxanthin appeared seemed to be positively influenced by *NCED.* As aril turning purple, the decreased expression of *CCoAOMT* seemed to facilitate the synthesis of apigenin. The substantial upregulation of *DFR* promoted the production of malvidin-3,5-di-O-glucoside. Additionally, the overexpression of MYBs may plays the important role in regulating the formation of different colored arils. In total, 14 genes were selected for qRT-PCR validation, the results indicated the reliability of the transcriptome sequences data. Our findings could provide valuable insight into the molecular breeding, development, and application of Maire yew resources.

## 1 Introduction

Fruit is an important vegetative organ of plants. Flesh and peel color are important trait that attract predators and enriches the horticultural crop (Wang et al., 2022). Fruit color are determined by pigment, of which flavonoids and carotene are the two most important pigments (Yu et al., 2022). Currently, transcriptome and metabolome analysis have been widely used to identify signal pathway and mechanisms that control pigment accumulation. This will provide the basis for gene mining and molecular genetic improvement of horticultural fruit color.

Flavonoids play a pivotal role in numerous biological processes, particularly influencing pigmentation factors and contributing to the diverse colors observed in peel, pulp, and flowers. (Ferreyra et al., 2012; Zhou et al., 2020). The biosynthesis pathway of flavonoids is relatively conserved. As an important catalytic enzyme in flavonoid biosynthesis pathway, phenylalanine ammonia-lyase (PAL) serves as a crucial catalyst in directing carbon flow into the phenylpropanoid pathway. The substrate catalyzed by chalcone synthase (CHS) is a precursor molecule for various classes of flavonoids. Flavonoid 3’-hydroxylase (F3’H) and flavonoid 3’,5’-hydroxylase (F3’5’H) actively contribute to the structural diversity observed within flavonoids. Dihydroflavonol 4-reductase (DFR) catalyzes the conversion of substrates that serve as precursors for the formation of pigmented anthocyanins (Li et al., 2023). For example, cinnamate 4-hydroxylase (*C4H*), *CHS*, and Glutathione S-transferase (*GST*) genes were strongly regulate the accumulation of naringin chalcone and anthocyanins in the red peel of *Vitis vinifera* ‘E-L 38’ (Ju et al., 2023). The activation of early phenylpropanoid biosynthesis genes, such as *PAL*, *C4H*, and 4-coumarate:CoA ligase (*4CL*), was more responsible for anthocyanin accumulation of *Solanum melongena*, and *F3’5’H* gene was positively correlated with the synthesis of delphinidin 3-glucoside in purple peel (Zhou et al., 2022).

Meanwhile, carotenoids serve as crucial secondary metabolites and stand as key indicators of fruit quality. (Luan et al., 2020). For example, carotenoids represent the pigments synthesized during the ripening process of fruit, specifically contributing to the eventual red hue observed in tomato (Perveen et al., 2015). Carotenoids, including lycopene and a range of alpha, beta, gamma, and delta carotenoids, undergo synthesis from isoprenoids (IPPs) via enzymatic catalysis. The accumulation of beta-carotene, violaxanthin palmitate, and rubixanthin laurate in Eriobotrya was regulated by the phytoene synthase (*PSY*), Zeta-carotene desaturase (*ZDS*), and zeta-carotene isomerase (*ZEP*) genes (Su et al., 2023). The *PSY*, Lycopene cyclase/phytoene synthase (*LCYE*), and *ZDS* were the key genes that regulated the lycopene accumulation of *Persimmon* fruits (Ye et al., 2022). Moreover, transcription factors (TFs) like myeloblastosis (MYB), basic helix-loop-helix (bHLH), and WD-repeat (WD40) are pivotal in regulatory processes. For instance, in apples, the *MdMYB10* transcribes and regulates the expression of the red skin color (Jia et al., 2020). *MYB* and *bHLH* genes were differentially expressed in red, yellow, and purple color of wolfberries (Duan et al., 2023). While the theoretical frameworks of flavonoid and carotenoid biosynthesis pathways have been established through existing research, further exploration is warranted to elucidate the mechanisms across different species.

Maire yew (*Taxus mairei*) is a rare and endangered species that belongs to the genus *Taxus* in the family of Taxaceae (Fu et al., 1999). The species has attracted much attention due to taxol, an anti-cancer drug it contains, and the high aesthetic timber (Huang et al., 2013). Moreover, Maire yew has a beautiful tree shape and bright red arils against with green leaves, which also is the perfect ornamental species used in landscape. Recent years, we have discovered interesting variations in aril color of Maire yew, including yellow and purple aril fruit in the field surveys. However, there have been limited insights on the aril color formation of this species (Cao, 2019), particularly the mechanisms of three different color formations.

To better understand the mechanism of color formations in Maire yew arils. In this study, three Maire yew germplasms with red (RTM), yellow (YTM), and purple (PTM) arils were selected in three different development stages. Integrative analysis of transcriptome and metabolome data were performed, our aims were to illustrate (1) the dominant metabolites of three different color arils in Maire yew; (2) the functional structural genes and TFs involved in flavonoid and carotenoid biosynthesis; (3) the metabolic pathways and the mechanism of color formation in Maire yew arils. These results could provide valuable insights for the identification of metabolites and candidate genes involved in pigment formation and it will accelerate the breeding process of Maire yew.

## 2 Materials and methods

### 2.1 Plant materials

**Figure 1:**
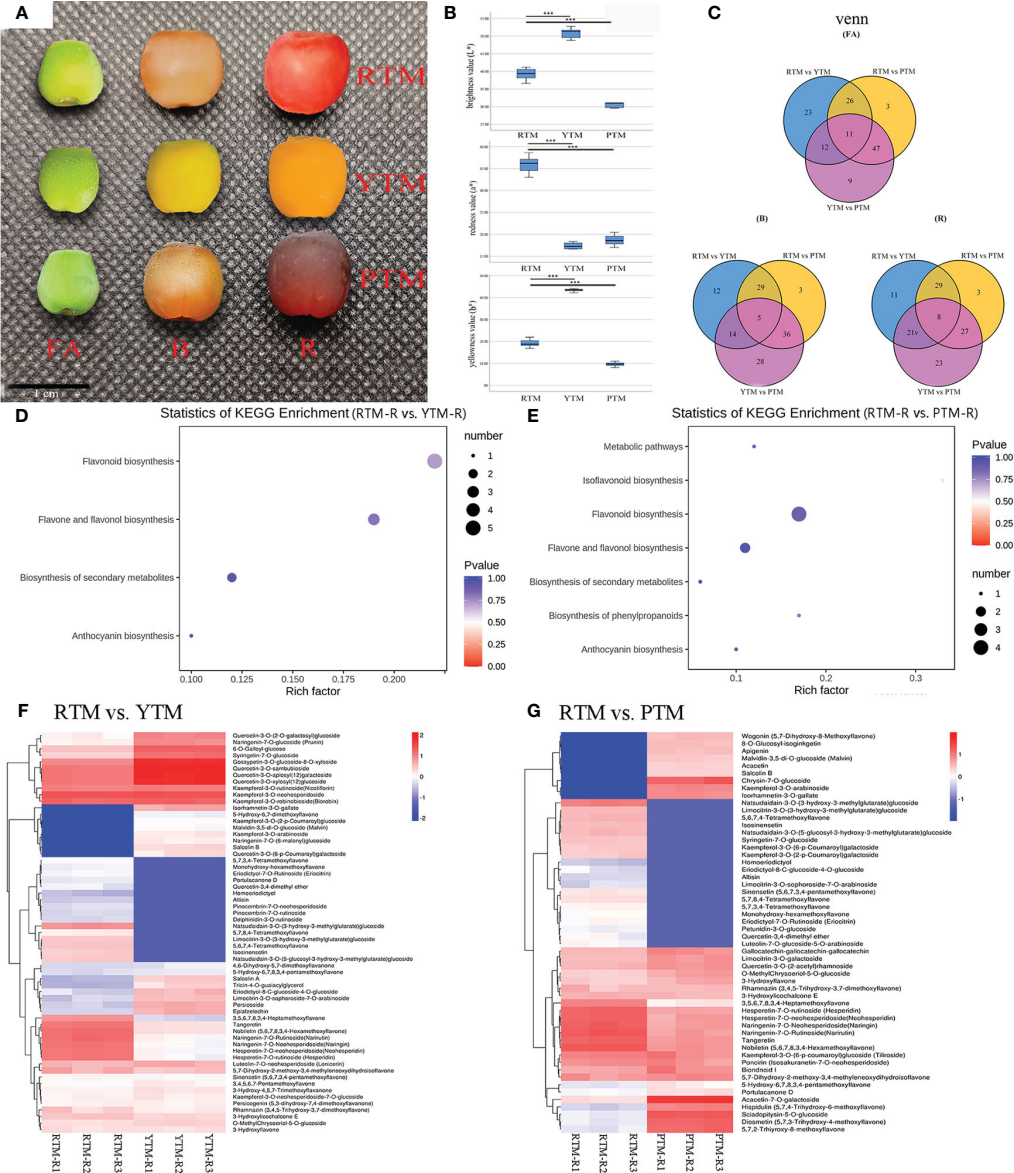
**(A)** The phenotypes of the fruit arils of *T. mairei*; red aril (RTM), yellow aril (YTM), and purple aril (PTM). Bar = 1 cm. **(B)** The aril color parameters; the L* means lightness. The a* represents redness or greenness. The b* represents yellowness or blueness. **(C)** The Venn analysis results of DAMs revealed by different comparisons. **(D)** KEGG enrichment analysis of the DAMs in RTM-R vs. YTM-R. **(E)** KEGG enrichment analysis of the DAMs in RTM-R vs. YTM-R. **(F)** Differentially accumulated metabolites in the pathway of flavonoid biosynthesis in RTM-R vs. YTM-R. **(G)** Differentially accumulated metabolites in the pathway of flavonoid biosynthesis in RTM-R vs. PTM-R. *** Represents a very significant correlation at the 0.001 level.

Three Maire yew samples with red (RTM), yellow (YTM), and purple (PTM) arils were collected with permission from the *Taxus* germplasm bank in Chenzhou, Hunan, P. R. China (lat. 25° 17′ 43″ N, long. 112° 40′ 22″ E; altitude, 294 m) (**Figure 1A**). In *Taxus*, the aril develops *de novo* as a collar at the base of the ovule. Then, it becomes visible as a green leafy structure (LA), which thickens and becomes fleshy (FA). The aril gradually develops a reddish color (B) and becomes bright and evenly distributed at the ripe stage (R) (Lovisetto et al., 2012). Here, fresh arils without seeds were collected at FA, B, and R stages. Rinsed with distilled water to remove surface moisture, each stage had three biological replicates and at least 15 fruits were mixed in each replicate. Three fruit per replicate were used for phenotypic measurements and pigment determination. Other samples were promptly frozen in liquid nitrogen, and subsequently stored at -80°C for subsequent metabolomic and transcriptomic sequencing analyses.

### 2.2 Measurement of Maire yew aril color traits

The color phenotypes of Maire yew aril samples (RTM, YTM, and PTM) at different stages (FA, B, and R) were measured using a color difference meter (CM-700d, Konica Minolta, Japan) to obtain the lightness value (L*), and hue value (a* and b*). Each epidermis parameter of arils was measured five times with three biological replicates. The L* parameter represents the lightness index, with higher values indicating lighter colors. The a* parameter represents redness or greenness, with positive values indicating redness and negative values indicating greenness. The b* represents yellowness or blueness, with positive values indicating yellowness and negative values indicating blueness. One-way ANOVA analysis was conducted by IBM SPSS 23.0 (IBM SPSS Software Inc., San Diego, California, United States). Statistical significance of differences of values were assessed with a Tukey’s test: *p < 0.05, **p <0 .01.

### 2.3 Identification of flavonoids and carotenoids

The metabolomic analysis of flavonoids and carotenoids in the Maire yew arils were conducted by three different aril samples (RTM, YTM, and PTM), three development stages (FA, B, and R), and three replicates by Metware Biotechnology Co., Ltd. (Wuhan, China). The freeze-dried arils were crushed in the mixer mill (MM 400, Retsch). To determine the type and content of flavonoids and carotenoids, samples were extracted with 70% methanol acetone solution and a blend of n-hexane, acetone, and ethanol (1:1:2, v/v/v) with 0.01% BHT (g/mL), respectively. The sample extracts were then subjected to Ultra Performance Liquid Chromatography-Tandem Mass Spectrometry (UPLC-MS/MS) analysis using a UPLC-ESI-MS/MS system (UPLC, SHIMADZU Nexera X2, https://www.shimadzu.com.cn/; MS, Applied Biosystems 4500 Q TRAP (Yu et al., 2020).

The linear ion trap (LIT) and triple quadrupole (QQQ) scans were acquired using a triple quadrupole linear ion trap mass spectrometer (QTRAP MS). The metabolites were detected and analyzed using a triple quadrupole linear ion trap mass spectrometer with electrospray ionization (ESI) and multiple reaction monitoring (MRM) mode. The MRM experiment was performed in triplicate to ensure the accuracy and reproducibility of the results (Chen et al., 2013). Principal component analysis (PCA) was carried out by FactoMineR package in R to characterize the accumulation patterns of metabolites across different samples (Zhang H. et al., 2022). Differentially accumulated flavonoids (DAFs) were identified based on the thresholds of variable importance in the project (VIP) value ≥1 and the fold change (FC) value ≥2 or ≤ 0.05. All DAFs were annotated using the KEGG compound database (http://www.kegg.jp/kegg/compound/). The metabolites were annotated by KEGG pathway enrichment analysis (http://www.kegg.jp/kegg/pathway.html), and the significance of metabolite-enriched pathways was determined by the hypergeometric test’s *P*-value.

### 2.4 RNA-seq library construction and sequencing

To perform transcriptome sequencing, a total of 27 libraries were constructed representing three different aril samples (RTM, YTM, and PTM), three development stages (FA, B, and R), and three replicates. RNA extraction was carried out using the Spin Column Plant Total RNA Purification Kit and following the manufacturer’s protocol. The purity of the extracted RNAs was assessed using 1% agarose gels. The integrity of RNA were evaluated using the Qsep1 instrument (Li X. et al., 2018). 1.0 μg of total RNA was utilized to construct the RNA libraries by the VAHTS mRNA-seq V3 Library Prep Kit. The library construction and sequencing procedures, including polyA-selected RNA extraction, RNA fragmentation, random hexamer-primed reverse transcription, and 150 nt paired-end sequencing, were executed using the Illumina HiSeq X-ten at Igenebook Biotechnology Co., Ltd.

### 2.5 Gene expression level and differential expression analysis

Raw data were filtered using Cutadapter (version 1.11) (Martin, 2011) to remove low-quality reads, characterized by those with over 50% bases,and a Q-value ≤20, and those with more than 5% ambiguous nucleotides. RNA quantification was performed using the Qubit RNA Assay Kit in Qubit 2.0 Fluorometer (Life Technologies, Carlsbad, CA, USA). RNA integrity was checked by the RNA NanoDrop 2000. The hisat2 software (Kim et al., 2015) was used to align the reads to the genome (downloaded from https://www.ncbi.nlm.nih.gov/assembly/GCA_019776745.1#/st). Gene expression analysis including transcript abundance estimation and normalization of expression values into FPKM (Fragments per kilobase of transcript per million fragments mapped), was conducted using the Trinity platform. The clustering heatmap was plotted using the Pheatmap package. The differentially expressed genes (DEGs) were identified by DESeq2 with the thresholds of adjusted p-value<0.05 and |log2Fold Change|>1 (Liu et al., 2021). All the DEGs underwent functional annotation and enrichment analyses. Gene Ontology (GO) and KEGG enrichment analyses with DEGs were performed using the GOseq package based on hypergeometric test (Young et al., 2010). The TFs prediction was carried out using the iTAK v1.6 software (Zheng et al., 2016). The Pearson correlation coefficient of the DEGs and DAMs was calculated using the cor function in R. The gene connection network was drawn with Cytoscape software version 3.10 (Su et al., 2014).

### 2.6 Quantitative real-time polymerase chain reaction validation

The qRT-PCR analysis was performed using the Universal SYBR Green PCR Master Mix Kit (Beijing Lanjeke Technology Co., LTD.) on the Roche Lightcycler96 Fluorescent Quantitative PCR System (Jimenez et al., 2018). 14 genes were selected for qRT-PCR validation based on their significant differential expression. The *GAPDH* gene served as an internal control (Wang H. et al., 2020). The specific primer pairs were designed using the Primer 5.0 software (Dossa et al., 2018), and they were listed in **Supplementary Table S1**. The relative expression levels of the selected genes were normalized to the expression level of the ACTIN gene (Cao et al., 2017). All the experiments were performed in triplicates, and the relative gene expressions were calculated using the 2^−ΔΔCt^ method (VanGuilder et al., 2008).

## 3 Results

### 3.1 Differences in color traits of red, yellow, and purple Maire yew arils

Collected samples of RTM, YTM, and PTM were measured at the ripe stage (R) to assess their aril color traits. Comparisons of different colored arils indicated that the negative b* value of RTM was significantly higher than that of YTM and PTM. The positive L* value was ranked in an ascending order of YTM > RTM > PTM. Compared to YTM and PTM, RTM exhibited a notably greater negative a* value. The variation trend of the b* value was similar to that of the L* value, namely YTM > RTM > PTM (**Figure 1B**, **Supplementary Table S2**).

### 3.2 Identification of the differentially accumulated metabolites of red, yellow, and purple Maire yew arils

Three types of Maire yew arils (RTM, YTM, and PTM) with various flesh colorations were collected at different development stages (FA, B, and R). Principal component analysis showed a high degree of aggregation among all samples (**Supplementary Figure S1**), indicating that the samples were highly repeatable and the data quality was reliable. Using UPLC-MS/MS analysis, a total of 266 flavonoids and 35 carotenoids were identified from all extracts of RTM, YTM, and PTM (**Supplementary Table S3**). Among these, flavones, flavonols, and dihydroflavones were found to be the most abundant flavonoid components.

Metabolites with significant differences between the three developmental stages (FA, B, and R) were selected. The fold change represents the ratio of accumulation levels between two samples. There were 72, 60, 67, 87, 73, and 67 significantly changed metabolites observed in the six comparison groups, corresponding to RTM-FA vs. YTM-FA, RTM-B vs. YTM-B, RTM-R vs. YTM-R, RTM-FA vs. PTM-FA, RTM-B vs. PTM-B, and RTM-R vs. PTM-R, respectively (**Figure 1C**). The DAMs between the six comparison groups are listed in **Supplementary Table S4**. A Venn diagram analysis revealed that 11, 5, and 8 compounds were common to the three stages (**Figure 1C**). In RTM-R vs. YTM-R and RTM-R vs. PTM-R comparison, the flavonoid biosynthesis pathway showed the most significant enrichment, with 17 (**Figure 1D**) and 11 DAFs (**Figure 1E**), respectively.

In YTM, the delphinidin-3-O-rutinoside, is an anthocyanin, and its accumulation over 10-fold lower than RTM in all stages. While the contents of isorhamnetin-3-O-gallate were 13.70- and 13.17-fold higher than RTM in the B and R stages, respectively (**Figure 1F**, **Supplementary Figure S2**). YTM displayed low levels of lutein, while its levels of Lutein dimyristate were higher than RTM which exhibiting a small accumulation in the three stages (**Supplementary Figure S3**). In PTM, the contents of chrysin-7-O-glucoside was increased (over 10-fold) than RTM throughout the coloring period. Meanwhile the contents of malvidin-3,5-di-O-glucoside (anthocyanin) was 8.97-fold higher than RTM in the R stage (**Figure 1G**, **Supplementary Figure S2**). Additionally, a small amount of neoxanthin and violaxanthin laurate were accumulated in PTM in the R stage (**Supplementary Figure S3**).

### 3.3 Analysis of differentially expressed genes in red, yellow, and purple Maire yew arils

To further investigate the molecular basis for flavonoid and carotenoid biosynthesis, a transcriptome analysis was conducted. Each sample was replicated three times, with independent library construction and sequencing. In this study, two principal components, PC1 and PC2, were extracted, accounting for 58.35 and 17.03%, respectively (**Supplementary Figure S4**). The transcriptome sequencing of the 27 aril samples resulted in a total of 175.97 Gb of raw reads. The raw reads were deposited in the National Center for Biotechnology Information (NCBI) (accession number: PRJNA1003858). After filtering the raw read for quality, 172.63 Gb of clean data were obtained, with 94.65% of bases scoring Q30. The GC contents of each sample ranged from 43.73 to 46.36%. Approximately 82.64-90.94% of the clean reads in the expression profile data of each sample could be mapped to the reference genome (**Supplementary Table S5**). These results indicate that mapped reads can be directly used to calculate gene expression.

**Figure 2:**
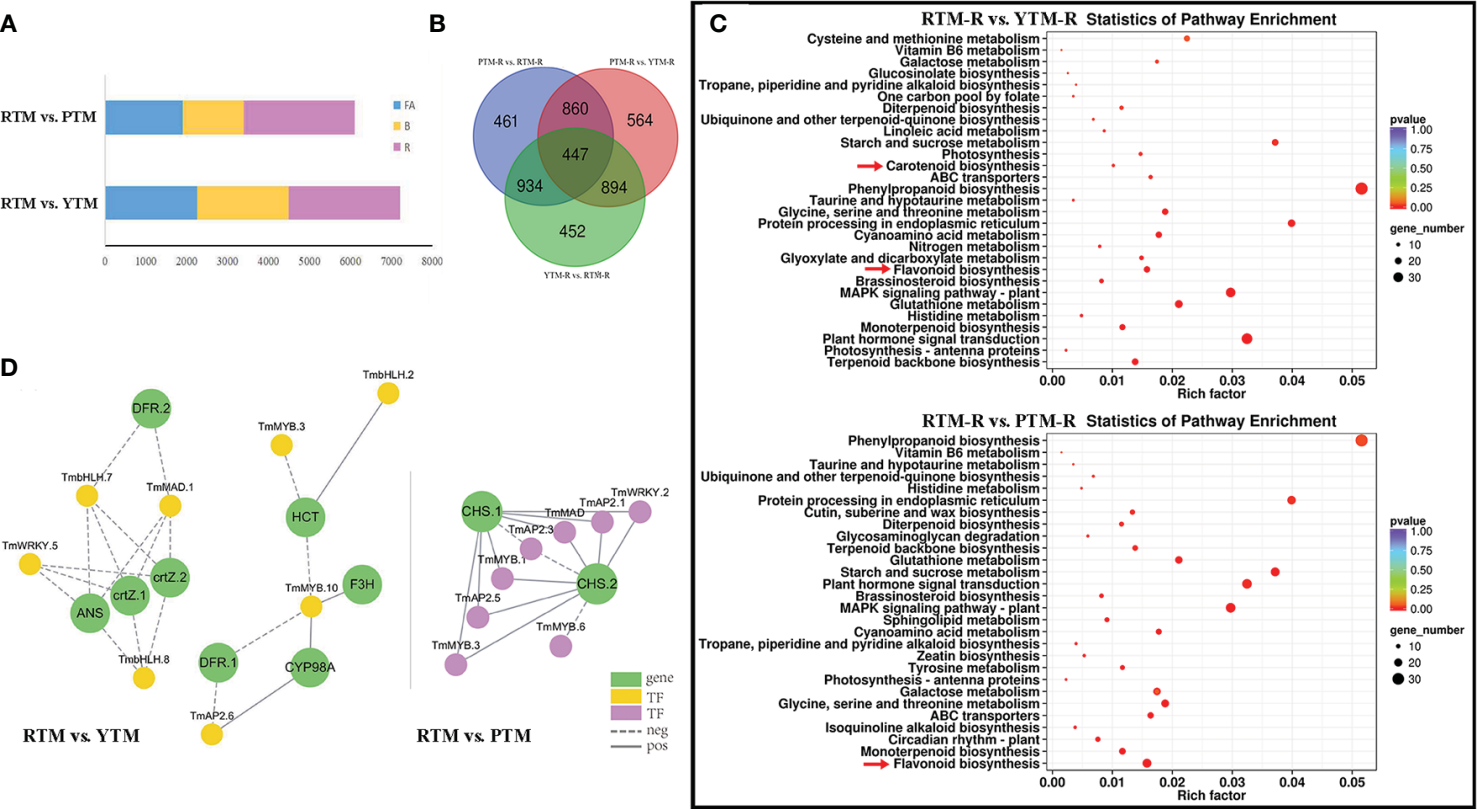
**(A)** The number of DEGs of RTM vs.YTM and RTM vs. PTM during three developmental stages. **(B)** The Venn analysis results of DGEs revealed by different comparisons of ripe arils. **(C)** KEGG enrichment analysis of the DEGs in comparison groups; the color and size indicate the p-value and the number of DEGs, respectively. **(D)** Regulatory networks of TFs and candidate genes; each yellow and purple circle represents flavonoid pathway genes and each green circle represents candidate genes.

To gain better understanding of the dynamic changes in gene expression during fruit ripening, we performed a kinetic analysis using the FPKM data from RTM, YTM, and PTM. Among the comparisons made between YTM and RTM, the largest number of DEGs was observed in RTM-R vs. YTM-R (2,727), followed by RTM-FA vs. YTM-FA (2,262) and RTM-B vs. YTM-B (2,229). Similarly, in PTM vs. RTM, the largest number of DEGs was found in RTM-R vs. PTM-R (2,702), followed by RTM-FA vs. PTM-FA (1,907) and RTM-B vs. PTM-B (1,492) (**Figure 2A**). These findings suggested that the fruit ripening stage should be targeted to get insights into the altered pathways and genes related to the flesh coloration of arils in Maire yew. Notably, the number of DEGs in the different color groups at the same developmental stage was greater than those in different stages of the same colored arils. Compared with upregulated DEGs, there were more downregulated DEGs in all comparison groups (**Supplementary Figure S5**).

We also compared the DEGs identified in RTM-R vs. YTM-R, RTM-R vs. PTM-R, and YTM-R vs. PTM-R, and drew a Venn diagram between comparison groups (**Figure 2B**). We identified 1,077 up- and 1,650 downregulated DEGs in comparison between YTM-R and RTM-R. Similarly, 1,165 up- and 1,537 downregulated DEGs were found in RTM-R vs. PTM-R. A GO analysis was performed to identify the biological processes that may be directly involved in aril color changes. These DEGs were primarily annotated in 15 biological process categories (BPs), 11 cellular component categories (CCs), and 4 molecular function categories (MFs), respectively (**Supplementary Figure S6**). The enrichment of specific pathways in different comparison groups were examined. The results showed that carotenoid biosynthesis (ko00906) and flavonoid biosynthesis (ko00941)) pathways were significantly enriched in the RTM-R vs. YTM-R analysis, suggesting their involvement in the color change from yellow to red (**Figure 2C**). Similarly, the flavonoid biosynthesis (ko00941) pathway was significantly enriched in the RTM-R vs. PTM-R analysis (**Figure 2C**).

### 3.4 Transcription factors analysis of red, yellow, and purple Maire yew arils

TFs play a crucial role in regulating the synthesis of plants and affect the color of fruit arils. In addition to structural genes, TFs associated with metabolite accumulation were vital in regulating the overall aril coloration. A comparison of YTM vs. RTM revealed differential expression of 141 TFs (38 upregulated and 103 downregulated). Among them, 1 *AP2*, 1 *C2H2*, and 1 *bHLH* genes were significantly upregulated, whereas 5 *MYB*, 10 *AP2*, 4 *WRKY* and 4 *bHLH* genes were significantly downregulated (P<0.05,∣logFC∣>2). Similarly, when comparing PTM with RTM, 147 TFs showed differential expression. There were 6 *MYB* genes (1 up- and 5 downregulated), 10 *AP2* genes (2 up- and 8 downregulated), 1 *C2H2* gene (upregulated), 2 *WRKY* genes (downregulated), and 2 *bHLH* genes (1 up- and 1 downregulated) exhibited significant differential expression (P<0.05,∣logFC∣>2, **Supplementary Table S9**).

Pearson correlations were calculated between key TFs and structural genes (**Supplementary Table S10**). With r > 0.8 and p < 0.001, the connection networks were shown in **Figure 2D**. Notably, the *ANS* and *CHS* genes were found to be highly associated with TFs, suggesting that they may serve as key regulators in yellow and purple arils, respectively. These findings indicate that these TFs could contribute to flavonoid and carotenoid biosynthesis in Maire yew arils.

### 3.5 Integrative analysis of transcriptome and metabolome

A total of 5 DAMs and 21 DEGs were co-enriched in the comparison between YTM-R and RTM-R in flavonoid biosynthesis (**Supplementary Table S6**). The epiafzelechin was identified as the crucial pigment for yellowness in YTM. The shikimate O-hydroxycinnamoyltransferase (*HCT*) was positively correlated with the accumulation of epiafzelechin (|Correlation| = 0.83). Meanwhile, the low expression of bifunctional dihydroflavonol 4-reductase (*DFR*), leucoanthocyanidin reductase (*LAR*), and anthocyanidin synthase (*ANS*) may play a crucial role in the substantial decrease of delphinidin-3-O-rutinoside. In RTM-R vs. PTM-R, 4 DAMs and 23 DEGs were co-enriched (**Supplementary Table S7**). Apigenin accumulates strongly in mature purple arils. The caffeoyl-CoA O-methyltransferase (*CCoAOMT*) genes were identified as crucial gene (|Correlation| = 0.85). There was a notable increase in the activity of *DFR* genes, leading to a substantial rise in malvidin-3,5-di-O-glucoside levels. The expression patterns of these genes may regulate the changes in aril color in Maire yew.

In carotenoid biosynthesis, we found 2 DAMs and 16 DEGs that were co-enriched in RTM-R vs. YTM-R (**Supplementary Table S8**). The accumulation of lutein is controlled by the low expression of *crtB* gene and high expression of *NCED* gene(|Correlation| = 0.83, |Correlation| = 0.83). Meanwhile, the *NCED* gene positively regulate the accumulation of β-Cryptoxanthin as the arils become yellow (|Correlation| = 0.80). However, there was no co-enriched gene found in PTM vs. RTM.

### 3.6 Quantitative real-time polymerase chain reaction analysis

To validate the reliability of the transcriptome data, 14 genes were selected for qRT-PCR validation based on their significant differential expression. Linear regression analysis demonstrated a good fit between the Log_2_(FPKM) values obtained from the transcriptome data and the Log_2_(2^−ΔΔCt^) values obtained from the qRT-PCR data, with an R^2^ value of 0.8296 (**Supplementary Figure S7**). The quantitative validation were in agreement with the expression trends observed in the RNA-seq data (**Supplementary Figure S8**). This indicated the reliability of the transcriptome sequences data in our study.

## 4 Discussion

### 4.1 Flavonoids and carotenoids are the main pigments in the arils of Maire yew

The phenotypic diversity of fruit is one of the most important traits for ornamental plants (Guo et al., 2021; Ji et al., 2021). Maire yew is an endangered and beautiful ornamental tree with red aril fruit against green leaves. The discovery of mutants with different aril colors of Maire yew provides a good subject for fruit color formations study. In our study, three Maire yew samples with red (RTM), yellow (YTM), and purple (PTM) arils were selected. The observed color distinctions primarily arise from the presence of specific pigments. To investigate the alterations in metabolite associated with aril color, we employed UPLC-MS/MS analysis. Further, through KEGG analysis, we discerned significant enrichment within the “flavonoid biosynthesis” and “carotenoid biosynthesis” pathways. Flavonoid biosynthesis is a well-studied secondary metabolic pathway in plants, known to be involved in the production of various pigments (Grotewold, 2006; Wang et al., 2023). Carotenoids, on the other hand, have received substantial research attention in horticultural crops, such as loquat (Zou et al., 2020), corn (Wang et al., 2020), and mango (Petry and Mercadante, 2016). In our study, a total of 267 flavonoids and 35 carotenoids were identified. Throughout the three coloring period, there were 11 and 1 common DAMs (over 10-fold) in YTM vs. RTM and PTM vs. RTM, respectively (**Supplementary Table S4**). These metabolites were likely contributors to the observed color variations.

The red color of Schizanthus grahamii’s petals was mainly supplied by delphinidin 3-O-rutinoside (Alcalde-Eon et al., 2013). In our study, we observed a significant down-regulation of delphinidin-3-O-rutinoside in YTM compared with RTM, which may contribute to the diminished intensity of its red color. Epiafzelechin and lutein were also identified as the dominant factors in yellow pigments (Lin et al., 2015; Lv et al., 2023). Similarly, our findings indicate a buildup of epiafzelechin and lutein in the YTM arils upon reaching maturity. The malvidin-3,5-di-O-glucoside was the main color factor in purple Shamrock (Luo et al., 2022). In the ripe aril of PTM, the content of malvidin-3,5-di-O-glucoside was 8.97-fold higher than RTM. Thus, malvidin-3,5-di-O-glucoside may be associated with the purple color observed. We also found apigenin accumulated in PTM arils at maturity, which may only be co-coloration with malvidin-3, 5-di-o-Glucoside, as it had been reported to play a co-chromatism in tan leaves of *Sorghum bicolor*. (Kawahigashi et al., 2016). The combination and accumulation levels of these compounds likely play a crucial role in determining the visible colorations in Maire yew arils.

### 4.2 Differential expression gene underlying the color-transition of Maire yew

In exploring the factors underlying the development of yellow and purple arils in Maire yew, we conducted differential expression gene (DEG) analysis specifically targeting the flavonoid and carotenoid metabolism pathways. In the ripe aril of YTM, the low expression level of *DFR*, *LAR*, *ANS* play a crucial role in the significant reduction of delphinidin-3-O-rutinoside, which were consistent with the studies of *Solanum melongena* and *Trifolium repens* (Zhang et al., 2022; Ma et al., 2022). In the synthesis of lutein, our findings indicate an increase in the *NCED* gene. Similar regulatory effect of *NCED* was reported in *Edgeworthia chrysantha* (Zhou et al., 2023). For PTM, the *DFR* gene showed a high expression level, which may regulate the accumulation of malvidin-3,5-di-O-glucoside in ripe arils. Martens et al. (2010) also found that the *DFR* gene can catalyze the conversion of dihydroquercetin to anthocyanidins. Additionally, the *CCoAOMT* gene was always associated with phenolic compounds (Yang et al., 2019), which in our study may reverse regulate the accumulation of apigenin. Metabolite analysis indicated that flavonoids and carotenoids constituted the primary pigments present in Maire yew arils. Variations in the expression levels of pertinent genes across distinct developmental stages ultimately account for the diverse coloration observed in Maire yew arils.

### 4.3 Transcription factors associated with flavonoid and carotenoid biosynthesis

Studies on ornamental plant species showed the that regulatory role of TFs in governing the expression of structural genes (Jochen et al., 2007; Yan et al., 2021). These regulators are pivotal in orchestrating the synthesis and accumulation of pigments responsible for fruit coloration. Specifically, MYB and bHLH TFs are involved in the activation or repression of genes associated with pigment biosynthesis pathways (Yu et al., 2020; Zhang et al., 2020). Most crucially, the positive or negative regulation of downstream accumulation is mediated by the MBW complex (MYB-bHLH-WD40) (Gil-Muoz et al., 2020; Zhou et al., 2022). In our case, a total of 141 TFs were identified, with 38 exhibiting upregulation and 103 displaying downregulation in the comparison between yellow and red arils. Similarly, in the comparison between purple and red arils, 147 TFs were identified, among which 85 were upregulated and 62 were downregulated. Among them, we found one *MYB* gene (KI387_007149) that was strongly expressed in yellow arils, suggesting its regulatory function in controlling the expression of key structural genes involved in producing the red phenotype. Furthermore, two *MYB* genes (KI387_015702 and KI387_022184) were overexpressed in purple arils, displaying close association with the *CHS* gene. Similar findings have been reported for the regulatory *PavMYB10.1*, which may determine the skin color patterns in *Prunus avium* (Jin et al., 2016). These suggest that the *MYB* gene may play a key role in fruit coloration, as recently reported (Ying et al., 2019; Liu et al., 2020).

## 5 Conclusions

Maire yew is a visually appealing ornamental tree in landscapes, the color variation of its arils is rich. In this study, we conducted metabolome and transcriptome analyses of three different color arils. A total of 266 flavonoids and 35 carotenoids were identified. High expression level of epiafzelechin, lutein, β-Cryptoxanthin and low expression of delphinidin-3-O-rutinoside were detected in YTM. And the high expression level of apigenin, malvidin-3,5-di-O-glucoside were found in PTM. These DAMs were crucial for the formation of arils color. Furthermore, 2,727 and 2,702 DEGs were obtained in RTM-R vs. YTM-R and RTM-R vs. PTM-R, respectively. The *HCT*, *NCED*, *DFR*, *LAR*, *ANS*, and *crtB* genes were closely related to the abundance of key metabolites in YTM. As the arils became purple, the *DFR* and *CCoAOMT* genes were identified as key factors. The overexpression of MYBs may plays an important role in regulating the formation of different colored arils. Our findings will provide valuable insights into the genetic mechanisms underlying aril coloration in Maire yew that can be utilized in genetic engineering or improvement efforts aimed at enhancing ornamental qualities.

## Data availability statement

The datasets presented in this study can be found in online repositories. The names of the repository/repositories and accession number(s) can be found in the article/**Supplementary Material**.

## Author contributions

YY: Formal analysis, Writing – original draft, Writing – review & editing. YFW: Conceptualization, Supervision, Writing – original draft. YW: Formal analysis, Writing – original draft. XW: Formal analysis, Writing – original draft. XL: Formal analysis, Writing – original draft. CW: Formal analysis, Writing – original draft. YZ: Formal analysis, Writing – original draft.
