# Supplementary figures and images for: Metabolome integrated with transcriptome reveals the mechanism of three different color formations in Taxus mairei arils
Source: Front Plant Sci. 2024 Jan 23;15:1330075. doi: 10.3389/fpls.2024.1330075 (PMC10844565; doi:10.3389/fpls.2024.1330075)

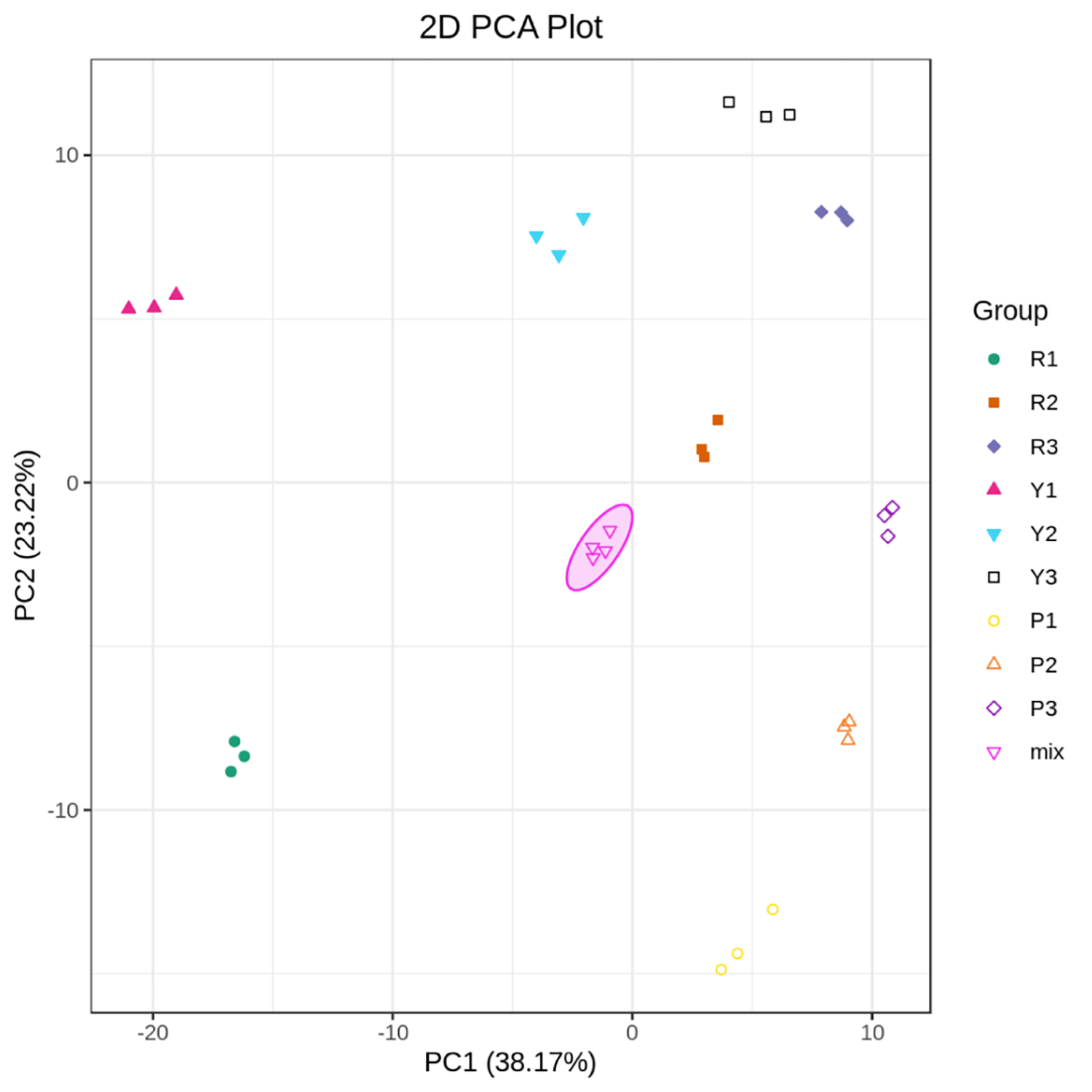

Supplement: Supplementary file 1 [file DataSheet_1.zip › Supplementary/Figure S1.tif]

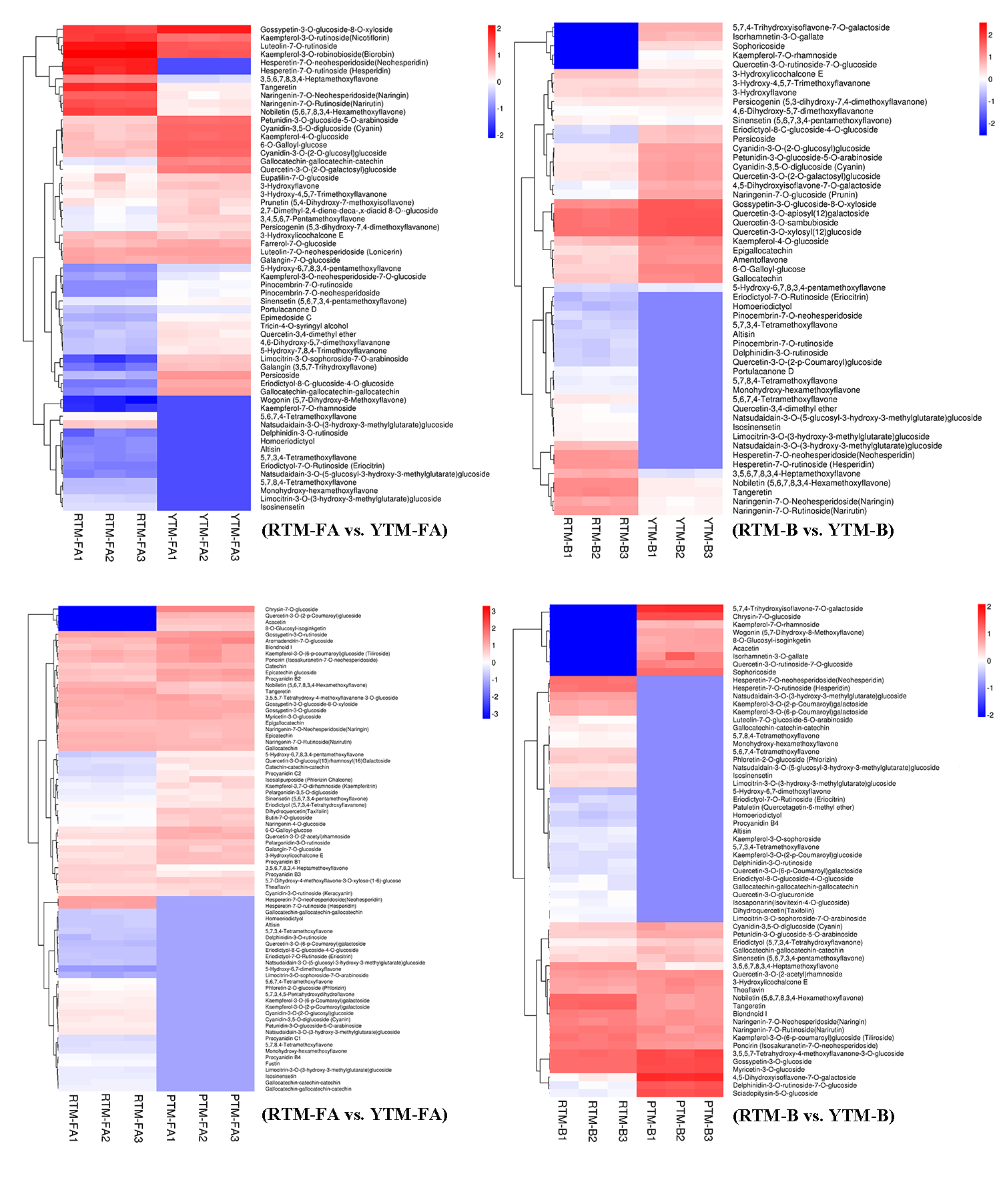

Supplement: Supplementary file 1 [file DataSheet_1.zip › Supplementary/Figure S2.tif]

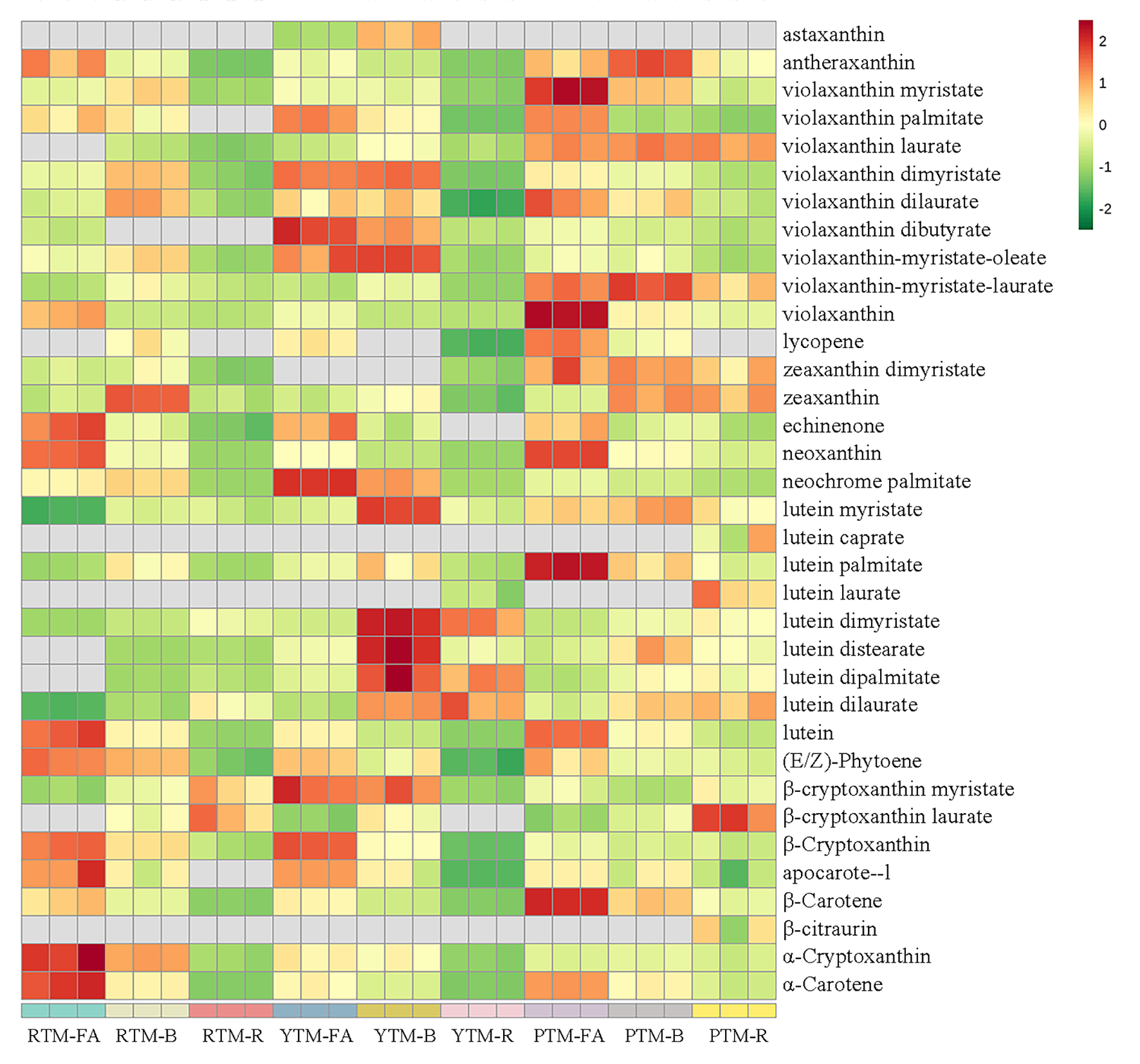

Supplement: Supplementary file 1 [file DataSheet_1.zip › Supplementary/Figure S3.tif]

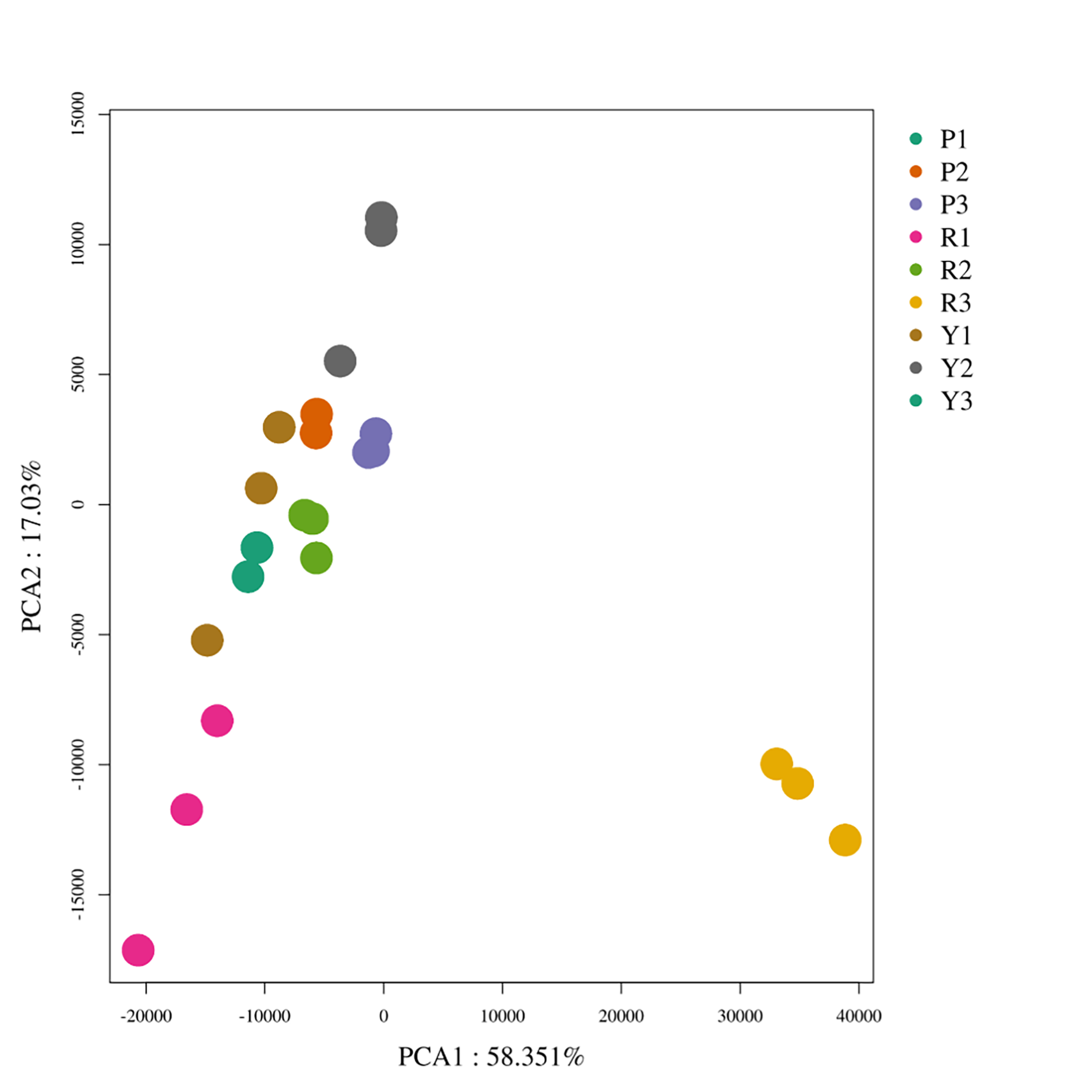

Supplement: Supplementary file 1 [file DataSheet_1.zip › Supplementary/Figure S4.tif]

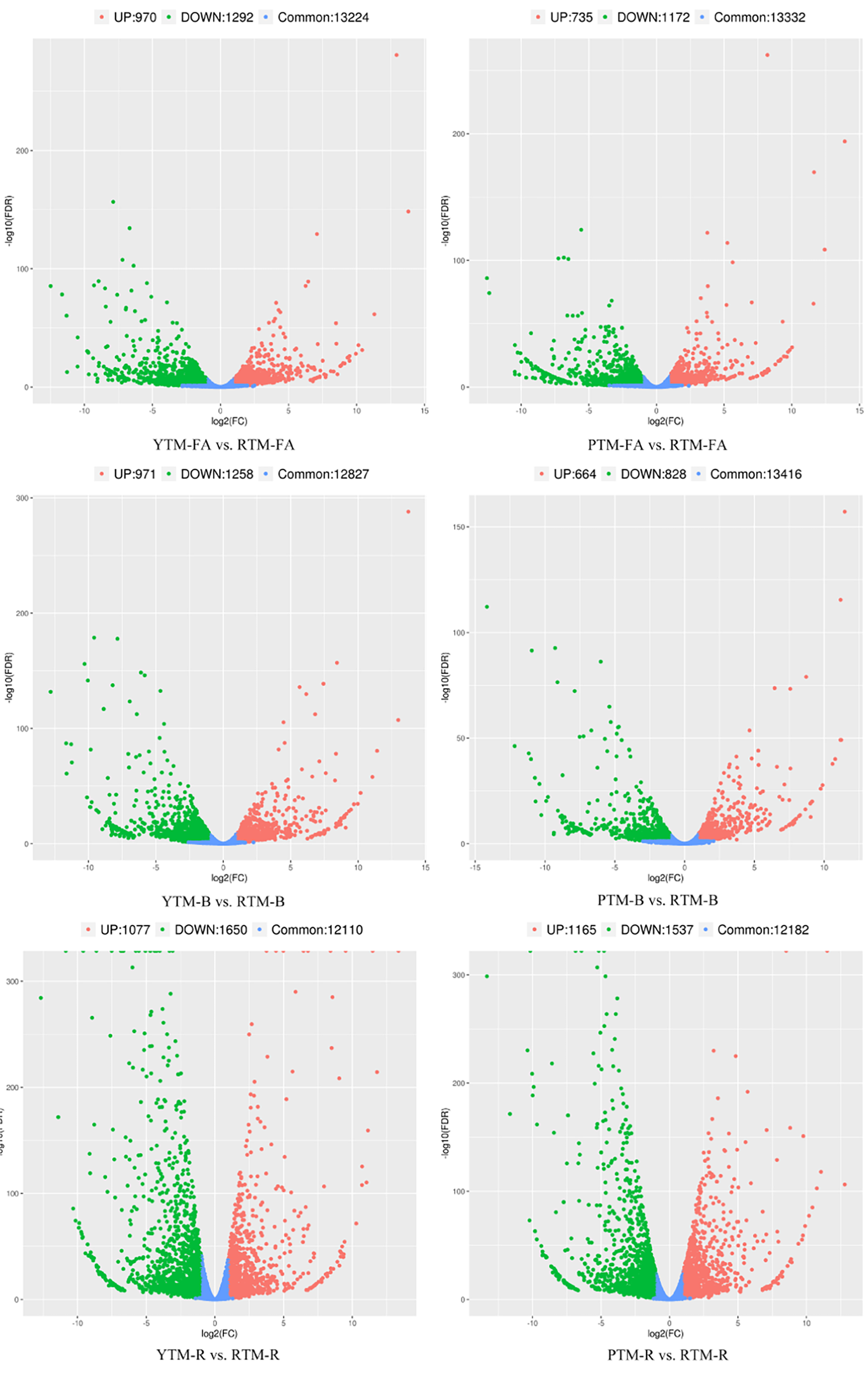

Supplement: Supplementary file 1 [file DataSheet_1.zip › Supplementary/Figure S5.tif]

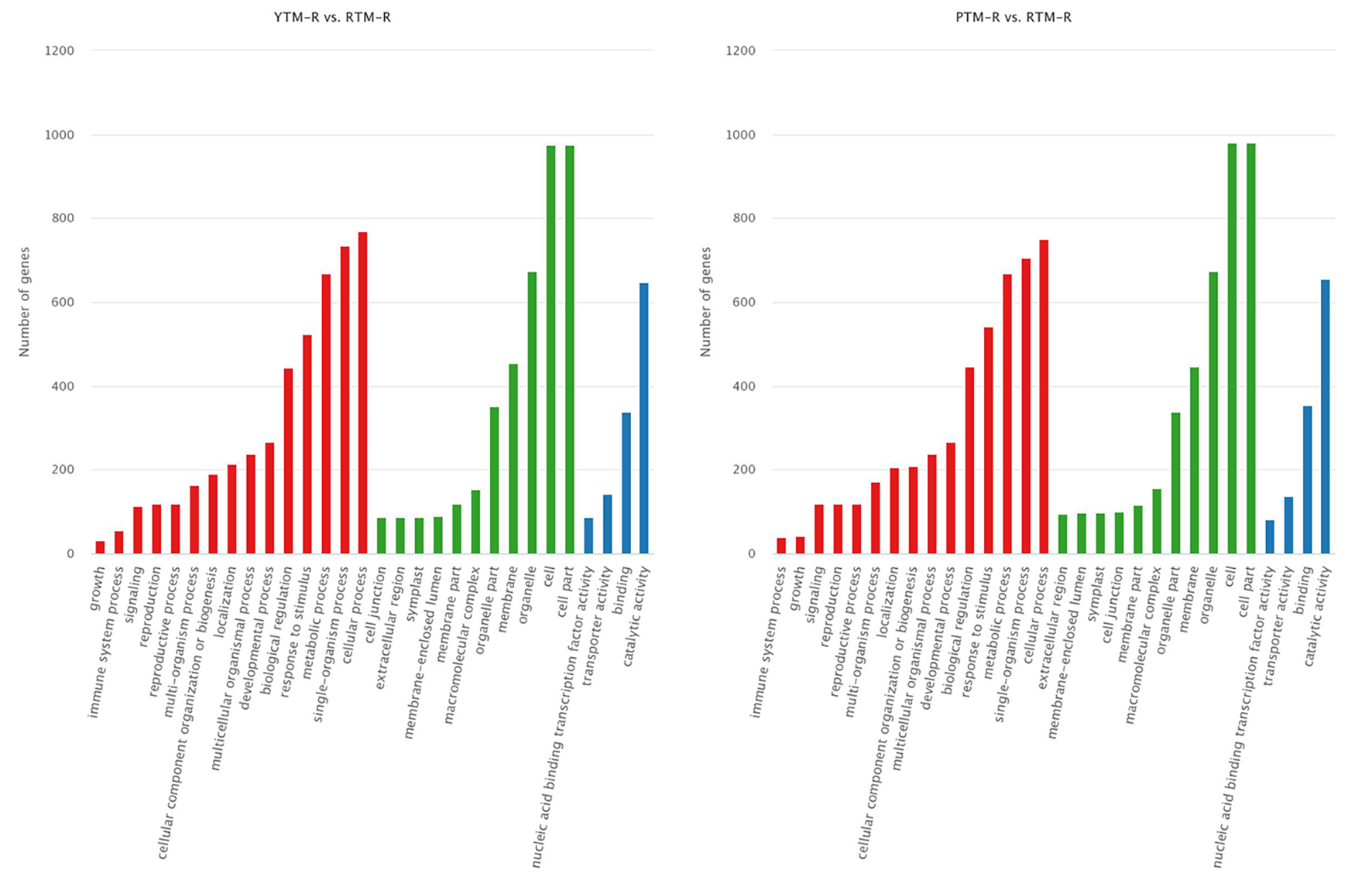

Supplement: Supplementary file 1 [file DataSheet_1.zip › Supplementary/Figure S6.tif]

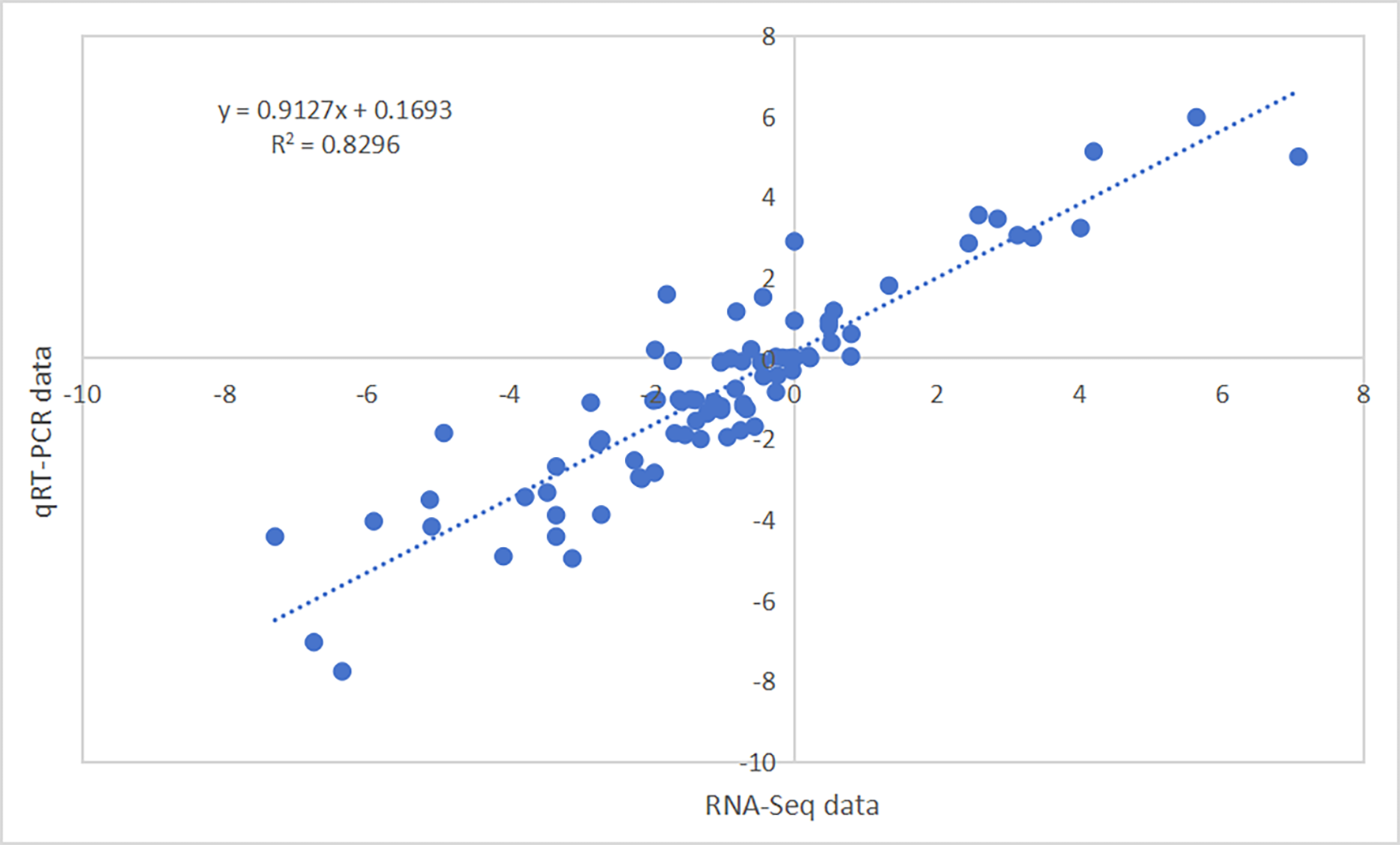

Supplement: Supplementary file 1 [file DataSheet_1.zip › Supplementary/Figure S7.tif]

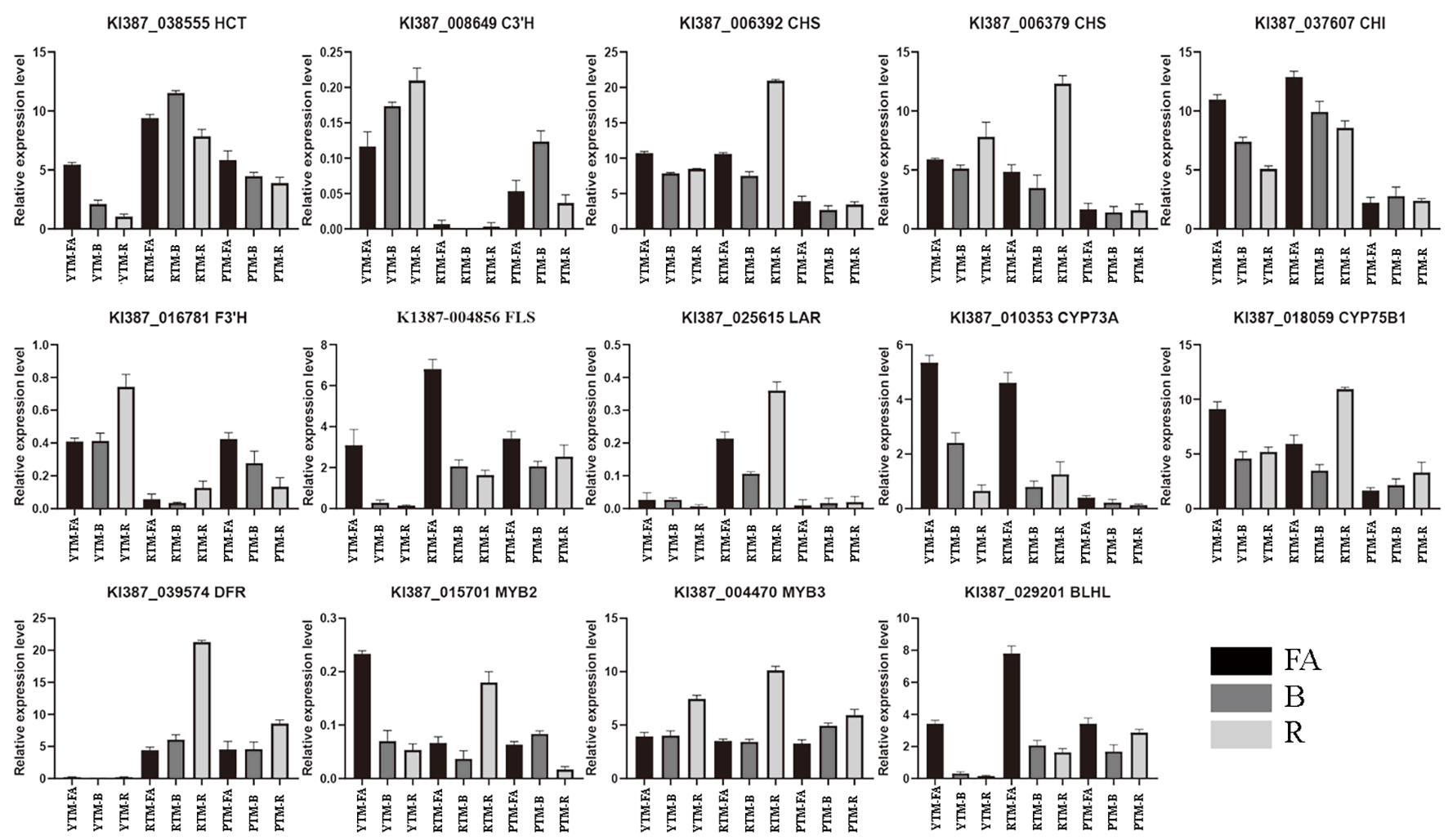

Supplement: Supplementary file 1 [file DataSheet_1.zip › Supplementary/Figure S8.tif]
